# "The Hospital" Nursing Mirror

**Published:** 1900-04-21

**Authors:** 


"The Hospital" Nursing Mirror.

Being the Nursing Section of "The Hospital."

[Contributions for this Section of "The Hospital" should be addressed to the Editor, The Hospital, 28 & 29, Southampton Street, Strand, London, W.C., and should have the word "Nursing" plainly written in left-hand top corner of the envelope.]

### Notes on News from the Nursing World.

### THE QUEEN AT A DUBLIN HOSPITAL.

One of the events of Tuesday in Dublin was the visit by the Queen to the Adelaide Hospital in Peter's Street, a very poor part of the city. Her Majesty was received with unbounded enthusiasm. Miss Fitzpatrick, the matron, who was attended by the sisters and nurses, had the honour of presenting the Sovereign with a bouquet; and in accepting it, the Queen said, "I hear you have a very beautiful hospital."

### TWENTY MORE NURSES FOR THE FRONT.

The exodus of nurses for South Africa continues. On Saturday the following twenty members of the Army Nursing Service Reserve left Southampton in the "Canada" for Cape Town: Misses C. L. Agg, G. A. Boyce, M. A. Cain, E. E. Cockburn, H. M. Curtis, M. G. Denton, M. T. Enright, M. C. Fraser, E. L. R. Ferguson, M. Goodline, E. A. Hancock, H. J. Henderson, A. D. Hook, M. G. Jones, A. V. Latham, F. Macdonald, A. N. Martin, J. Overbeck, M. Patteson, and C. L. Travis. Of these nurses Miss Maria Theresa Enright was trained at St. Bartholomew's Hospital, and was subsequently staff nurse in the same institution. Since November, 1895, she has been attached to the Nurses' Co-operation, New Cavendish Street. Miss Edith A. Hancock was trained at St. Bartholomew's Hospital and the Royal Infirmary, Derby. From September, 1897, to April, 1898, she was attached to the Brooklyn Institute, Anerley, and since that time has been sister at the Ear and Throat Hospital, Birmingham. Miss Flora Macdonald was trained at Wandsworth and Clapham Infirmary. She has since been staff nurse in the same institution; assistant nurse at the City Hospital, Birmingham; and private nurse at the Nurses' Institute, Sheffield, and the Nurses' Institute, Halifax. From 1895 she has been attached to the Scarborough Nurses' Co-operation. The ten nurses of the Welsh Hospital sailed in the same vessel.

### THE NURSES OF THE SCOTTISH NATIONAL RED CROSS HOSPITAL.

At Rosneath, on Saturday, Princess Louise presented the nurses who are going out with the first section of the Scottish Red Cross Hospital with their badges as members of the Army Nursing Reserve. The nurses are Miss Sharman (superintendent), and Nursing Sisters Alexander, Trout, Ritchie, Thomson, Storrie, Murdoch, and Forbes. After the ceremony of presenting the badges had taken place, her Royal Highness and the Marquis of Lorne showed the party over the perfectly appointed and charming convalescent home which they have prepared at Rosneath for returned invalid soldiers, and afterwards entertained them to luncheon. The complete equipment and full staff of the first section of the hospital sail for the Cape on Saturday next by ss. "Pembroke Castle" from Southampton.

### THE RETURN OF MISS McCAUL.

Among the passengers who arrived at Southampton on Saturday in the "Dunvegan Castle" were Miss McCaul and Mr. Treves. Miss McCaul, who enjoys the distinction of having accompanied a field hospital, and of being the first nurse to enter Ladysmith, has gone into the country for the rest which she needs after her exciting experiences. It is reassuring to learn from Mr. Treves that our wounded in South Africa have been well looked after. The testimony of an expert who has been three months on the spot is worth all the assertions of people whose statements are founded on hearsay.

### AUSTRALIA'S LOYALTY.

An Australian Matron writes: "I cannot describe the unbounded enthusiasm which has held our great continent of Australasia in its grip since the beginning of the war in South Africa. From every quarter, including New Zealand and gallant little Tasmania, offers of men, money, and horses have poured in. From the thousands of the millionaire to the modest penny of the schoolboy, all have contributed alike in response to the appeal from the mother-land. This is the more remarkable as the expression of feeling from a community which represents all nationalities. Yet here all are free alike, and because of that freedom they uphold the mother-land in her determination to secure like liberty for other sections of the Empire. Hundreds of loyal hearts are tremulous with joy over the unity they had hoped for, but scarcely dared to believe could be so soon and so truly ratified, and many are the fervent prayers that the glorious bond so ratified may never be loosened. Sydney has given of her best in sending such representatives of the medical profession as Drs. M'Cormack, Liaschi, and Scot-Skirving, with many others. Fourteen of our best-known and trained nurses also accompanied the Army Medical Corps of New South Wales, and, with every modern appliance and all that skill and knowledge can do, will doubtless prove of immense value at the front. Nurses are offering eagerly for active service, and the great difficulty is to choose the most suitable where there is such abundance of material from which to select. Already £60,000 has been subscribed in New South Wales alone, and those not able to give money or men are sending comforts of every kind."

### A GERMAN TRIBUTE TO BRITISH NURSES.

Dr. Krummacher, one of the German medical attachés who has just returned from South Africa, and has been describing the details of the British medical service to a Berlin audience, referred in terms of warm admiration to the Army Nursing Sisters. He expressed his regret that such an institution as an organised corps of nurses belonging to the army is unknown in Germany, where the nurses in time of war are supplied by the religious communities and by the Red Cross

Association. Imitation is the sincerest form of flattery, however, and as Dr. Krummacher finds so much that is good in our system, it ought not to be impossible for him to persuade the military authorities in his own country to organise one on similar lines.

### THE ST. PATRICK SISTERS.

It is announced that the Duchesse di Villanda is organising "a corps of lady helps" to aid the wounded at the front. In these circumstances it is perhaps necessary to warn any ladies who think of joining that if they go to South Africa without the consent of the War Office they will not be permitted to undergo "the rough experiences of camp life" upon which the Duchesse dilates. Moreover, instead of extending a hearty welcome to an unauthorised band of women, "in khaki jackets and skirts, with red collars and felt hats," Sir Alfred Milner will certainly include them in the category of visitors who are a positive nuisance.

### "DEAR NURSE."

It is marvellous how thrilling can be the adventures of an imaginative Red Cross nurse. A Mrs. Marie Thompson, much beloved by the people of Osney, Oxfordshire, who speak of her as "dear nurse," has, according to her own account, seen much of life since last October, when the war began. According to the account given in the *Oxford Times*, she states that she left Netley and sailed as a Red Cross nurse for Cape Town. On landing she went straight to the front, and was present at the battle of Colenso, being attached to the camp and not to the hospital! She alleges that she herself assisted to carry many of the wounded off the battlefield, eventually returning to England in charge of the invalids on board the "Princess of Wales" hospital ship. She contemplates returning again to South Africa as she has undertaken to purchase a wreath for the relative of an Oxford young man who died at Kimberley, and place it on his grave, unless circumstances intervene. Unfortunately she is now under remand on a charge of obtaining money by false pretences. In three respects, at all events, "Dear Nurse" has been romancing. She is not known at Netley, and is not, therefore, a Red Cross nurse; only two nurses, of whom Mrs. Marie Thompson was not one, have been allowed on the field; and no person of that name is attached to the nursing staff on the "Princess of Wales" hospital ship.

### NURSING BY THE HOUR IN NEW YORK.

A curious account of her experiences in hourly nursing is given by Miss Grace Forman, a graduate of the New York City Training School. She had cards printed and circulated among doctors and generally, to the effect that she was prepared to do hourly nursing as follows: Obstetrical cases, six hours or less, 3 dollars; remaining with patients all night, 3 dollars; surgical and obstetrical dressings, twice daily, 10 dollars per week; general cases, 1 dollar per hour. Miss Forman wrote on the cards, "Deserving cases cared for without charge during the summer"; and on the reverse side referred to twelve doctors who had given her permission to use their names. She has now been nursing in this way nearly a year, and states that she finds it far more satisfactory to both patient and nurse than she could have expected in her most sanguine moments. She has never been without a patient at any time, and has sometimes had more than she could do. There is no doubt that the work is very hard; and Miss Forman adds that "it is neither fun nor play to create a demand for a new thing, although it may be the very thing needed by half the public."

### THE IMPROVEMENTS AT THE MIDDLESEX HOSPITAL.

The many improvements both effected and now in progress at the Middlesex Hospital will vastly benefit the nurses. The staff has been increased in order to provide attendance for the patients in the new cancer wing, and there will be a further addition when the new nurses' home is built. The advance proof of the plans of the proposed home are prepared for the consideration of the governors. Three houses in Cleveland Street will be razed to the ground, and the home will be erected in their stead. Part of the site will be occupied by the laundry, but the greater portion is to be devoted to the home. The ground floor will be divided into a large sitting-room, a visitors' room, and four bedrooms with bath and lavatory accommodation. The first, second, and third floors are each arranged for eleven bedrooms. The strength of the staff will probably be augmented to the extent of from twenty to thirty nurses. The new nurses and servants' quarters in the cancer wing are exceptionally complete, the rooms are lofty and adequately furnished, the decorations cheerful and in good taste. The floors in each case are covered with cork carpet and a rug. Every nurse has a wardrobe, dressing-chest, washstand, easy chair, and a book-case; whilst the sitting-room is most comfortable, with Chesterfield couch, easy chairs, piano, book-case, occasional and writing tables. The servants' rooms are quite apart from those belonging to the nurses. There is a bath-room on every floor, and the walls are lined with white glazed bricks, which give a clean and wholesome appearance. The cancer wards are each served by three nurses, two on day and one on night duty. The whole staff is in charge of the matron, Miss Davies, under the supervision of Miss Thorold. There are several novelties in the ward of interest to nurses. The centre table, for instance, runs nearly the whole length of the room. On the top are flowers and pots, and beneath it is fitted with cupboards, one of which is allotted to each bed, the others being used for nursing requisites. One end of the table has a marble top for the hand basins. Each patient has also a bedside locker for her china, fork and spoon, &c., which are reserved for her exclusive use.

### NORTHAMPTON ARTISANS AND THE NURSING INSTITUTE.

It is very satisfactory to learn from the report of the Northampton District Nurses' Fund that the artisans in the town made a liberal response to an appeal for assistance on behalf of the new nursing institute. This was the first occasion on which they were asked to assist in raising funds, and the numerous employés in factories who gave up their Saturday half-holiday, or their earnings, to the work of collecting contributions merit warm acknowledgment. The scheme of the institute was launched in January, and the building, which is to cost £2,335, is now in course of erection. During last year upwards of 11,000 visits were paid to

486 cases. The accounts of the Northampton and County Nursing Institution, which are kept quite separate from those of the District Nurses' Fund, show that the nurses earned upwards of £800. Excellent reports have been received on the nurses' work from the medical men under whom they have been employed, and in two serious epidemics of diphtheria and typhoid, congratulatory letters have been sent to the committee from ten medical officers of health expressing their admiration of the manner in which the nurses had carried out the arduous duties under exceptionally trying circumstances. Altogether Miss A. F. Lunn, the lady superintendent, has every reason to be proud of the admirable way in which the obligations of the staff have been fulfilled, and of the appreciation of their services manifested by the residents.

### IMPROVING THE TRAINING OF WORKHOUSE NURSES.

In the ninth annual report of the Northern Workhouse Nursing Association, whose staff now consists of 83 nurses, it is a matter of comment that several of the training schools have decided no longer to train nurses for one year only, but have made two years the minimum of training. The committee point out that this means "a considerable increase in the cost of the training of each nurse, and that unless the subscriptions are increased they will be compelled, most reluctantly, to reduce the number of probationers at a time when their services are more and more required." Unfortunately, the deficit in the funds of the association is £175, as against £92 in the previous year, and an appeal for further financial support is therefore obviously needed. Although Poor Law nursing is still far from what it should be, some of the people in rural districts are still using the untenable argument that "what was good enough for our fathers is good enough for us," there is not the slightest doubt that such organisations as this are very helpful, and merit warm support. It is curious, by the way, to find that the Northern Association last year supplied nurses to workhouse hospitals in Basingstoke, Wimborne, Cheltenham, and Bridgwater, and we see that Cambridge and Suffolk have now been added to the list of counties in which its nurses are working.

### THE STAFFORDSHIRE NURSING INSTITUTION.

Progress continues to mark the career of the Staffordshire Nursing Institution. In the report for 1899, the twenty-eighth of its existence, the committee express the belief "that the reputation of the staff for skill and careful and painstaking attention to their duties has never stood higher." Another feature of the report is that the number of cases nursed is greater than that of any previous year, and the earnings are proportionately in excess. The figures show that 977 cases were nursed by 133 nurses, who earned the large sum of £5,800. It has for some time been the practice of the committee to divide among the staff the greater part of the balance standing to the credit of the institution at the end of the year. On this occasion the sum is £770, as compared with £465 last year. For the cases nursed gratuitously, or on reduced terms, £130 was drawn from the sick poor nursing fund, to the credit of which the annual subscriptions—£87—have been placed. A well-deserved compliment is paid by the committee to Miss Shirley, the lady superintendent. "It is owing," the report says, "to her experienced administration and her happy relations with her staff that the success of the institution is maintained."

### CREEDS ON COMMITTEES.

At the annual meeting of the Redditch Nursing Association a heated discussion arose on an issue which should never be raised on such occasions. Nursing organisations should have nothing whatever to do with the differences between Church people and Nonconformists. In this case the proposal that the committee should consist of an equal number of Churchmen and Nonconformists provoked some angry remarks, though as it appears that the donor of the nurses' home wished for the division suggested, one would have thought that his desire would have been discussed without lighting the fires of religious controversy. At the same time, members of a committee of a nursing home should not be selected because of their adhesion to any particular church or denomination, but solely on account of their business capacity, the interest they take in the work, and the possession of the necessary leisure to attend to it.

### SHORT ITEMS.

The annual financial report of the Harlech District Nursing Association, which was adopted the other day, is of a favourable character, thanks largely to a dance at Talsarnau, and concerts at Harlech and Llanbedr.—Miss Mary Wardell, whose Convalescent Home for Scarlet Fever at Stanmore, Middlesex, has such strong claims on public sympathy and support, will have a stall on behalf of the institution at the United Sale of Work in the Albert Hall, Knightsbridge, on Thursday and Friday, May 17th and 18th. Some of our readers may like to send Miss Wardell useful contributions.—Readers of the correspondence headed "Easy Swindles" in our columns, will learn with interest that Mary Beatrice Manning, who is charged with obtaining money from nurses and others under false pretences, has been committed for trial at the Central Criminal Court.—As usual, in view of catastrophes on Hampstead Heath on Easter Monday, the St. John Ambulance Brigade provided medical and nursing assistance, a doctor and five nurses being in attendance, with a waggon, a litter, and 70 men. There were several accidents, and their services were much appreciated.—Mr. Richard Greene will deliver the sessional lecture in connection with the Royal British Nurses' Association on "The Insane, the Asylum, and the Nurse," next Thursday evening at the Rooms of the Medical Society of London, 11, Chandos Street, Cavendish Square, at six p.m. The chair will be taken by Sir Henry Burdett.—It is anticipated that the "Maine" will arrive at Southampton on Monday next. Those wishing to send communications to friends on board should address them: "Hospital Ship 'Maine,' c.o. Executive Committee, 30, Curzon Street, W.," or to the secretary, Mrs. Blow, Walsingham House Hotel, Piccadilly, W. Such letters should be received prior to Saturday, the 21st inst.—The Lord and Lady Mayoress of Liverpool will hold a reception to the nurses of the hospitals and charitable institutions of the city and neighbourhood on April 30th and May 1st.

### Lectures on Medicine to Nurses.

By H. A. LATIMER, M.D. (Dunelm), M.R.C.S. Eng., L.S.A.Lond.; Consulting Surgeon, Swansea Hospital; Past President of the South Wales and Monmouthshire Branch of Brit. Med. Assoc.; Past President of the Swansea Medical Society; Hon. Life Member of the St. John Ambulance Association, &c.

### VI.—PAST TREATMENTS OF DISEASE.—SHIFTING THEORIES OF DISEASE.

ALTHOUGH it will be necessary to speak of special treatments of various diseases when I come to consider these in their order, there are certain general principles which hold good for application in all cases of departure from health. These are based upon the commonsense use of knowledge which has been gained as to the normal physiological action of the organs within our bodies, and as this knowledge has been enlarged and progress has been made in the study of the organism in health and in disease, there is little reason to doubt that those who are living in these days of enlightenment have a great advantage over those of an earlier generation. A short review of old ideas of the treatment of disease in the past cannot, I think, fail to be interesting to my readers, so I shall turn aside for awhile to consider this part of my subject.

I would have you know, first of all, that there are few callings where fashion reigns so supreme as it does in the profession of medicine. I will take for this word "fashion" a dictionary definition, which declares it to be a mode of action prevailing at a particular time. I take it that a particular line of thought concerning so experimental or conjectural a subject as that of the nature and treatment of deviations of health in the body is impressed upon the general body of practitioners of medicine from time to time by some original thinker, who succeeds in influencing others, either by his writings or by oral teaching, or sometimes by the success he obtains outside of the great body of those who practise the healing art. Certainly this has been very much the case in the past, schools of thought having arisen to sway, for a time, the general bulk of medical men; the ideas formulated by such teachers have often, in their time, given way to new ones which have been exposed, and these, again, have been displaced by newer ones. There is no finality in thought, and with new knowledge comes new theories concerning the nature and treatment of disease. Thus the methods advocated for the cure of disorders of the body are seen to have been the reflex of the ideas concerning the nature of these disorders when they were promulgated and adopted. When it was considered that insanity was the outward manifestation of the possession of the unfortunate lunatic's body and soul by evil spirits, it was not an unreasonable method of treatment when these last were taken to the priest, who would, by candle, bell, and book, and by a theory of transmitted spiritual authority, exorcise and drive away the intruders to the regions from whence they had come. Given beliefs that lunacy was of this nature, and that theological teachers possessed these powers, the logic of this plan of treatment during the prevalence of such beliefs was sound and rational. With the departure of belief in a spiritual possession of the lunatic by the evil powers, there still remained vague and unscientific theories concerning the nature of insanity. The brain as an organ of mind was out of court, and it was believed that the malady, or maladies, which constitute madness could be best treated by subjecting persons of unsound mind to harsh and cruel treatment. "Formerly a lunatic was regarded with horror as a being who had lost all relation to society, and was to be treated as a wild beast; he was confined in a gloomy, filthy cell, was loaded with chains, and shut out from all influences which could cheer his mind or lead it from the subject of its delusions." During the prevalence of such theories horrible cruelties were habitually practised on the insane until a light broke into the minds of humane men that the whole treatment so adopted was wrong and useless. Then, owing to the work of Dr. Pinel, in France, and of William Tuke, a Quaker, in England, sounder views on this subject began to prevail, and the mechanical restraint and cruel treatment of lunatics was considerably lessened. It was not, however, until the advent of Dr. John Conolly that the abominable system which had hitherto been in force was altered. Some relics of the old beliefs evidently linger amongst the community; for if insanity shows itself in a family, the subject is spoken of with bated breath, as if some disgrace attached to it. When the fact is fully realised that when any organ in the body becomes deranged the especial work which it has to perform is badly done, it will be seen that the manifestations of an unsound mind are simply due to perverted function and faulty working of the brain, which is the organ of thought. The laity well understand that if the stomach is disordered indigestion of food will follow; that if the bronchial tubes are inflamed cough and difficulty of breathing will ensue; that if muscular rheumatism sets in there will be pains and stiffness in the parts so affected; and that, in short, derangement in the health of any organ in the body will be followed by disturbance in the work which it is the peculiar duty of that organ to perform. When people will apply this knowledge to disturbances of function in the brain they will cease to regard insanity as an exceptional disease, and will recognise that its various manifestations are but the products of disturbances in the centres of reason, intelligence, memory, will, and self-control.

Reverting to the subject of fashions in medicine, it will be seen on examining those of the past and the present time that they have been, and are, but the expression of the knowledge possessed by people at the time they have obtained. As science is progressive, and as our means and instruments for research become improved and perfected, so theories shift to adapt themselves to newly-ascertained facts. Just as a man who desires to be a mechanical engineer sets himself to learn the component parts of machinery in order to understand how they should work correctly and evenly before he attempts to put right any defects which may become revealed by their being put into action, so it is necessary that the physician should study the anatomy of the human body, by which he learns the component parts of the human machine, and should then study physiology, which will instruct him in the work which is performed during the health of these parts. He will then find that he ascertains much of the ravages wrought on the organism by disease if he studies morbid anatomy, which reveals to him the changes which have been brought about by this agency. But it was just these very points which it was difficult to study in the past, for the community was unwilling to supply the "subjects" which were the necessary parts of the investigation. So men had to fall back upon theories of disease, which were the result of their imaginations rather than their knowledge. In such days what was called science was really, for the most part, empiricism. It was what Oliver Wendell Holmes in his "Professor at the Breakfast Table," calls "Pseudo-Science"—a pseudo-science being a "nomenclature, with a self-adjusting arrangement, by which all positive evidence, or such as favour its doctrines, is admitted, and all negative evidence, or such as tells against it, is excluded. . . . A pseudo-science does not necessarily consist, wholly, of lies. It may contain many truths, and even valuable ones. The rottenest bank starts with a little specie. It puts out a thousand promises to pay on the strength of a single dollar, but the dollar is very commonly a good one."

### Nursing at Maritzburg.

By an Alexandra Nurse.

The Camp, Fort Napier, St. Patrick's Day.

Our work goes on much in the same way here, so far as nurse and patient are concerned. There has been no fresh fighting for a week past, but invalids and the previously wounded are being transferred to the hospitals here. This week I have had many more patients to dress at our outdoor inspection-room. The number looked almost alarming on Monday morning to the surgeon-major and doctor who inspect the men. Some 200 appeared from Chieveley—tired, worn, begrimed, and travel-stained in appearance, and almost without exception requiring dressing and other attentions to wounds and bruises. These are my special care, and with the help of an orderly they were soon attended to. Those who needed were made comfortable with socks, for in many cases they had none ; so you may guess the state of their poor feet.

The wounds are very varied. Many have limbs and knees swollen from rheumatism ; three have synovitis ; and partial paralysis of parts following wounds is a very common thing. Several are dull of hearing from head wounds and the noise of the artillery combined. On Tuesday night another 170 arrived, also from Chieveley. Poor fellows ! They are very patient under all their hardships, and so grateful. This is St. Patrick's Day, and though a true Scot I am going to wear green belt and ribbons in sympathy with my many Irish patients. My surgeon-major and doctor are also Irish. To-day brought a young lieutenant with a very bad hand, the result of being shot at Colenso. One finger had been amputated, and on the back close by the thumb was a swelling or blister as large as an egg filled with green pus, evidently the result of a poisoned bullet.

Two more of my women left to-day for England. These have a heavy burden to carry, as in each case the husbands are invalided, the victims of nervous and mental prostration, and will be of little assistance to them in travelling. One has six children and the other two. This week the married sergeants were allowed to come down from the front for a three days' visit. You may imagine how excited the poor wives were, and no wonder, as six little strangers had come in the fathers' absence, and had to be presented for a first kiss from Daddy.

### A Soldier's Garden Party.

As you have been hearing so much recently of the trials and hardships of the boys at the front, I must tell you of a gleam of sunshine that came into the lives of not a few on Saturday last. By the kind invitation of Miss Browne, the lady warden, who is at the head of the Diocesan College of St. Anne, a large party of our sick and wounded convalescents were entertained to a concert and garden party in the college grounds. The soldiers, some ninety in number, walked slowly down from the wards of the lower barracks, under the guidance of two ward masters, who were most kind, gentle, and attentive to their feeble army. Six who are still using crutches were brought down in rickshas, but the college being very convenient to the camp, none were unduly fatigued. Four nursing sisters were also present, and rejoiced in seeing their patients so happy. Arriving punctually at three o'clock they were received by the Lady Warden at the outer entrance, who personally shook hands with and welcomed each man as he entered. A little further along, the pupils, a kindly and charming set of young ladies, pinned a button-hole on each man's jacket, which were of the bright or royal blue colour usually worn by military invalids. When all were comfortably seated in the large schoolroom an excellent programme was gone through, the lady teachers and pupils being the artistes. Solos, choruses, violin and pianoforte selections, a Japanese duet and dance, and, lastly, a skirt dance, kept the men in a state of delight for an hour. The college is a very quaint and picturesque building, and was at one time a residence for the Governor of Natal. It is built in the form of a square, with a large tennis court in the centre bordered by vines, bamboo, and yucca trees, and other tropical plants. A pomegranate tree has been my special admiration for many weeks. Its thick, dark branches, laden with large and crimson fruit, formed quite a picture. In this court chairs and lounges were placed for the men, and tables laden with delicacies were dotted all over. The young ladies poured out tea and waited upon the soldiers, also showing their sympathy by listening to the interesting details of how, when, and where they were wounded. To these dear girls their guests were heroes, and to the heroes the girls appeared as angels. Several snapshots were afterwards taken amidst much fun and laughter. Cigars and sweets were also liberally supplied, and after another hour thus pleasantly spent three cheers were given for our Queen, three more for the soldiers of the Queen, followed by three for the Lady Warden and Miss Moore, and so a very happy and memorable afternoon was brought to an end.

### The Nurses of the Imperial Yeomanry Hospital.

Writing from Deelfontein under date of March 25th, a correspondent says : On Saturday March 17th, exactly a month since our departure from England, we arrived at our destination, Deelfontein, or the Devil's Fount, celebrated in this part of South Africa for the abundance and purity of its water supply—a most important consideration in choosing a site for a large base hospital. Apart from the Fount, Deelfontein consists only of the station (with a post and telegraph office) and a store—a store that sells everything a white man doesn't want, except, I must not do that store an injustice, "Callard's Butter Scotch" ! The camp of the Imperial Yeomanry Hospital lies immediately on the Cape Town side of Deelfontein, and already presents a most striking appearance. Thanks to the energy and foresight of the commanding officer waterpipes have been laid on, and a siding constructed, so that Red Cross trains can run right up to the huts. The first thing that caught our weary eyes was the officers' mess tent, temptingly laid with dainty white cloths ready for luncheon. We had been prepared for "roughing" it, but that certainly was out of the question on the first day of our arrival. After luncheon we had a tour of inspection round our premises. Next to the officers' mess tent is the only brick building on the place, labelled Commandant's office, and outside it is the Imperial Yeomanry Hospital post-box. Then come the ward huts and tents. The huts are very light and airy, and each holds 20 beds (these being, by the way, of the most modern "chain" type, the gift of manufacturers at home). The tents for the patients are most inviting looking of the tortoise type, each capable of holding eight to ten men. Then there are a few bell tents for use by the convalescents until the rest of the huts can be erected.

### The Sisters' Encampment.

Beyond the wards and tents is the "Sisters' encampment," which consists—or consisted on our arrival—of a mess tent, two huts, and three other tents for sleeping purposes. Sunday, however, proved to be a day of typical African wind and dust storms, and our tents collapsed. Such things, however, are a mere detail in camp life, and to the credit of the Kaffirs and the vigilance of the orderlies it must be said that none lost the most trifling article permanently, though of course many things were broken.

### Arrival of the Red Cross Train.

The next day proved to be our first "Admission Day," and naturally great excitement was felt at the arrival of the Red Cross train with some 110 patients for us, mostly transferred from the hospital at Naauwpoort. Everything was arranged in a most orderly way, and in a very few minutes the sick and wounded were comfortably housed in the huts and tents provided for them. They proved to be chiefly regulars, men of the Black Watch, Gordons, and Argylles, &c., with a sprinkling of Canadians and Australians, together with some C.I.V.'s, and later in the week several Yeomen were admitted. Of course, many and wonderful are the tales told of hardships and dangers cheerfully endured, and of brave deeds, some of which have won for the doers the honour of the V.C., and others equally brave that are only known to a few.

### The Love of "Bobs" and Chocolate Boxes.

But in all the tales the men tell two facts are brought into striking prominence—one is the great love that is felt by all troops for dear old "Bobs," and the other is the affection they have for their chocolate boxes, most of which have been carefully sent home. I heard one owner describe his as a "rare fancy thing." Needless to say, the comforts of the hospital are much appreciated. Men who have been for months "at the front," and have gone through all the hardships those three short words imply, are grateful for every little thing; and the generous public who have so thoughtfully supplied this as well as other hospitals with such things as tobacco, writing materials, handkerchiefs, &c., &c., may rest assured that they have done much to relieve the hard lot of the suffering soldiers.

### Guarding the Camp.

The safety of the camp is looked after through the day by a number of orderlies told off to act as policemen, who, amongst other things, keep a watchful eye on the stores and prevent "commandeering"; and at night—when, by the way, the camp, with its rows of brightly-lit huts, has a most fascinating appearance—by sentries supplied by the military camp, which is about a quarter of a mile away. These sentries add much to the amusement of the inhabitants by most religiously challenging all passers-by, even though they wear an apron and a red cross. It makes one realise forcibly that the country is in reality under martial law, and that it is a grave reality and no child's play. This fact is, of course, brought home to one in many ways—by the constant passage of troops of all sorts up country (Driefontein being on the main line) with their horses and equipment, as well as by the carefully-guarded prisoners on their way to Cape Town and St. Helena.

### The Nurses' Bookshelf.

[We invite Correspondence, Criticism, Enquiries, and Notes on Book likely to interest Women and Nurses. Address, Editor, The Hospital (Nurses' Book World), 28 & 29, Southampton Street, Strand, London, W.C.]

The Nursing Profession: How and Where to Train. Being a Guide to Training for the Profession of a Nurse, with Particulars of Nurse Training Schools in the United Kingdom and Abroad, and an Outline of the Principal Laws Affecting Nurses, &c. Edited by Sir Henry Burdett, K.C.B., author of "Hospitals and Asylums of the World," &c. Second Year. (London: The Scientific Press, Limited. Price 2s.)

Nowadays, when so many young women of all ranks and degrees of fitness want to become nurses, a handbook like this is invaluable. It will at once show the aspirant and her friends where she may get the training she needs, and under what conditions as to age, payment, and the like—points which are generally asked about, and on which the vaguest replies are given by friends and acquaintances. While this part of the volume is of most use to the prospective nurse, the other, which gives details of such parts of our law as may at one time or another in her career affect a nurse, are of the highest importance to everyone engaged in the work. Hardly any nurse of experience has escaped being placed in positions when she would have been glad to know what statements on her part were privileged, and what would render her liable to an action for slander. She may also occasionally want to know when she can raise an action of slander against someone who has been speaking ill of her. With regard to the notification of diseases, also, the registration of deaths, and similar points, occasions arise when a nurse's acquaintance with the demands of the law may be of use to the ignorant and distracted relations of the patient. All this information the nurse will get in this handy little book. And, when so many nurses are going abroad, the lists of training schools and nursing institutions abroad, both in our colonies, in the United States, and on the Continent, will be found very helpful. It is a pity that no information is given as to the rates of pay, and, if possible, the prospects of employment for private nurses abroad; but perhaps this hiatus may be filled up in some succeeding edition, though the editor is wise to give no information if he cannot obtain that which is reliable. This is a thoroughly useful book, which meets an acknowledged want.

The Art of Feeding the Invalid: "A Series of Chapters on the Nature of Certain Prevalent Diseases and Maladies, together with Carefully Selected Recipes for the Preparation of Food for Invalids." By a Medical Practitioner and a Lady Professor of Cookery. New and popular edition. (London: The Scientific Press, Limited, 28 and 29, Southampton Street, Strand, W.C. Price, 1s. 6d.)

The success of this little book is shown by the fact that a new edition has been called for. In it the word "invalid" is taken in the largest sense, and advice is given as to the best kinds of food for many chronic conditions, as well as for cases of acute disease. The medical practitioner who has collaborated in the compiling of the volume is, we presume, responsible for the selection of the kinds of food suitable for patients during the course of febrile diseases and in convalescence, in indigestion, gout, liver disease, diabetes, and consumption, and for the dieting that will best meet the requirements of sufferers from corpulence, constipation and diarrhœa, and chronic invalidism. He gives the names of the various foods permissible under different conditions, while his lady collaborator, in the latter part of the book, gives full and very clear instructions for preparing them. The dishes include a large number not usually associated with invalid cookery, but this makes them all the more valuable in a work of this nature, as so many people seem to think that an invalid must always be fed on the most tasteless and monotonous diet, and it is quite worth while to know what things of a more savoury nature are wholesome, or at any rate not injurious in the various diseases dealt with. In short, this little book, clear, scientific, practical, and cheap, is well worth possessing by every mistress of a house, while it is invaluable to the matrons and housekeepers of every hospital or home where the sick are treated. We commend it most cordially.

### The English Hospital, Haifa.

By a Special Correspondent.

THE DAY'S WORK.

Perhaps some idea of an ordinary day's work in an Eastern hospital may interest nurses in the West, and other parts of the globe. I will try to give an example of the usual routine. We get up at six o'clock in the morning; have breakfast—after having previously given the patients theirs —at seven a.m., and begin the day with service at the little English church adjoining the hospital, half an-hour later. At eight o'clock we go to the wards, and superintend the early morning's hospital work, which in any part of the world must be pretty much the same. Of course, we nurses wash and dress the women and make their beds. We also wash the men who are very ill, and only allow the ward man to make the beds of the mild "cases." Next come whatever dressings have to be done. Anytime after nine o'clock we may expect either our German medical man or the native doctor. The latter speaks French, and always writes his prescriptions in that language. About ten a.m. the patients have their lunch, and after that time, unless a new patient arrives or something unusual has to be done, we feel that our charges are settled for the morning. One of us then betakes herself off to the kitchen to superintend the cooking. The dispenser, when not at the town dispensary, will probably do some hospital dispensing, see to her stores, &c. The third nurse will devote herself to some of the many odd jobs which accumulate everywhere. Just before noon the patients have their dinner; ours follows. In the afternoon most of the natives, including our wardman, take a siesta. Beyond giving the after-dinner medicines, and the patients their three o'clock meal, unless someone is very ill, there is but little to do in the wards. At five p.m. we have evensong in church, and afterwards begins the second battle of the day, *i.e.*, the cooking of the evening meal for patients and nurses. Very often the cook lets the kitchen fire out while we are in church, and then despair overtakes her because she cannot get the meat brown in the oven. With regard to times off duty, we usually have, or try to have, afternoons and evenings alternately quite free. When there is night duty the hours for the night nurse are from nine p.m. to six or seven a.m. She generally goes straight to bed, and has her free time in the afternoon and evening.

Patients' Diet.

Patients who are on full diet have native food, viz., for breakfast—coffee and native bread; lunch—bread, olives, or oranges; for dinner—a native dish of meat, usually served with onions, and, of course, rice and bread. The afternoon meal is the same as their lunch; and for supper they have rice and leban. Leban is sour milk—a kind of curds and whey. It is supposed that Jael gave Sisera leban, when she "brought him butter in a lordly dish."

Our Town Dispensary.

Besides the dispensary at the ho[illegible], we have a large one in the town of Haifa, about a mile away. There, three times a week, out-patients are received and prescribed for by the native doctor. There is an English lady on our staff who is a qualified dispenser. She usually rides to the dispensary on a donkey, followed by a native servant with a basket of bottles. The dispensary is a most important and flourishing part of our work here, and the natives really seem to appreciate it.

General Hints and Impressions.

Life in an Eastern hospital is certainly interesting and instructive. Each day brings novel and unlooked for experiences. Any nurse who thinks of coming to Syria should be prepared for many vicissitudes, and always for the unexpected. She should be a broad-minded woman, and able to adapt herself to any circumstances. There is no "society"—except, perhaps, in Jerusalem and Beyrout—but the few English residents in the various centres are very friendly, and everybody is kind to the nurses. Anyone who appreciates an unconventional life would be happy here. The only recreation is riding, and a nurse who does not care about equestrian exercise would feel rather "out of it," and would, besides, lose many of the intensely interesting excursions which are to be made in Palestine.

The Influence of the Holy Land.

And, of course, this Land, with all its deep and holy associations, must always remain different from any other. Every spot is so full of history and thought. It is helpful, too, to think that the people in their manners and habits have altered so little during the past 2,000 years. At every turn one sees practical illustrations of the Bible, and each day one receives fresh and beautiful impressions; associations rush into the mind, connecting closely the past with the present. I think one's after-life can never be quite the same after having lived here—perhaps not quite so lonely. For, surely, when we are back in the rush and turmoil of the world, visions of the many restful scenes of Palestine will present themselves to our minds, and, somehow, things will be different. It is, certainly, a very great privilege to have sojourned awhile in this Holy Land, to which we all owe so much. All that English nurses can do to help the suffering natives, too, is but repaying one iota of the great debt. If it be permissible to say so, it seems that here, even more than elsewhere, "Ye did it unto Me" is being echoed in some region not very far off.

### Presentations.

Birmingham Skin Hospital.—Miss Smith, the matron of the Birmingham Skin Hospital, has been the recipient of several tokens of regard and esteem. A number of former patients, through Mr. T. W. Stanley, presented her with a diamond ring and a stationery cabinet, and from the nurses of the institution she has received a set of silver teaspoons.

Church Deaconnesses' Institution, Chester.—Nurse Nicholas and Nurse Robinson have been presented by the council of this institution with gold medals in commemoration of the fact that they have been attached to it for an unbroken period of twenty-one years.

### Where to Go.

Empress Rooms, Royal Palace Hotel, Kensington.—Monday, April 30th, three p m., cafe chantant in aid of the Indian Famine Fund. Tickets, 5s. each.

Stafford House, May 8th.—Concert on behalf of the Mary Wardell Home for Scarlet Fever.

### Death in Our Ranks.

Another Victim of Typhoid.

We regret to announce the death of Nurse Edith Dismore at the Fern Hospital, Mountain Ash. Miss Dismore, who was only 28 years of age, contracted typhoid fever whilst she was on duty as a member of the Cardiff Nurses' Co-operation, and succumbed to the disease. The superintendent says that she was an unselfish and conscientious worker, and spent her life willingly in the service of others.

### The Princesses at the Irish Hospitals.

BY A SPECIAL CORRESPONDENT.

Two Royal visits to hospitals took place in Dublin last week —Princess Henry of Battenberg's visit, with her children, to the Children's Hospital in Temple Street, and Princess Christian's visit to the City of Dublin Hospital, for the purpose of laying the foundation stone of the new nurses' home.

At the Children's Hospital the Royal party were received by the Most Rev. Dr. Donnelly, Bishop of Canea, the Superioress of the Order, and a large group of well-known physicians, surgeons, and patrons of the hospital, which is under the care of the Sisters of Charity, assisted by a body of well-trained secular nurses, and an unusually able medical and surgical staff. The building is largely modern, though its basis is (or was) a fine old last-century mansion, and it is in every way up to date. Lately a new wing has been added, comprising waiting-rooms, office, children's wards, and accommodation for nurses. The number of intern cases treated last year was 154; extern, 3,328.

The Royal visitors were conducted round the wards, pausing frequently to talk to the patients, and to give them flowers. The Duchess of Connaught seemed especially at home with the little ones, who lost all shyness at the sight of her cheerful, pleasant, pretty face; but, of the Royal children, the two Battenbergs seemed to display most interest. The visit was not an especially formal one, the Princesses talking in a desultory and friendly way with patients, nuns, and doctors as they went over the whole of the large, handsome building.

The "Moy Mell" Children's Guild (named after the bazaar held for the hospital's benefit in 1898, and patronised by her Excellency Countess Cadogan) has for its object the making of children's clothes for the hospital and the supply of toys. The young Prince and Princesses inscribed their names as members of the guild before leaving the building. A short congratulatory address was read, and Princess Henry of Battenberg replied in a few kindly words.

The nuns in this hospital, as in others superintended by the nursing orders, supervise the wards, taking a large share in the training of the probationers (with the collaboration of the doctors and matron), and keeping always a kindly eye to the mental and physical welfare of the little waifs of humanity who fill the homelike wards. Although under Roman Catholic management, patients of all religions are received, and no interference whatever is made with their personal beliefs or customs.

Princess Christian's visit to "Baggot Street" Hospital (City of Dublin Hospital) was of a more formal character. The new nursing home is the outcome of a very radical change in the constitution of the hospital itself—which has been carried on for a long time under rather curious conditions. Until lately the nurses of the hospital were supplied by the City of Dublin Nursing Institute—a limited liability company, with premises across the road distinct from the hospital. This institute took (and still takes) probationers, giving them their training in many different hospitals, and using a special uniform—brown, with brown bonnet and veil, and badge of the City of Dublin "Three Castles." The City of Dublin Hospital, among others, obtained its nurses from the institute, which had all power of selection, refusal, examination, and certification, the hospital having no control whatever over its nurses, except during the hours they spent in the wards. They boarded, slept, and attended lectures in the institute. The matron and doctors of the hospital had practically no jurisdiction over the nursing staff. It was considered advisable to make some alteration in this arrangement, and sufficient money has therefore been specially collected to build a nurses' home in the hospital's own fine grounds, so that the nursing staff will in future belong solely to, and be trained solely by, the City of Dublin Hospital—known, since Princess Christian's visit, as the Royal City of Dublin Hospital. Pending the erection of the new building, the nurses sleep in special dormitories made out of the disused theatre buildings, which have lately given place to the magnificent new theatre block. They wear a black uniform, with small plain black bonnet; but no veil. They will receive all their training in the hospital, and be under the able control of Miss Shuter, the matron of the "Royal."

### Appointments.

MILDENHALL COTTAGE HOSPITAL.—Miss A. M. Kelly has been appointed Nurse-Matron. She was trained at the Nightingale Home, St. Thomas's Hospital, and has since been, in succession, sister at the General Infirmary, Worcester; district nurse at Greenhill, Harrow; and senior sister at the Cardiff Sanatorium.

FEVERSHAM COTTAGE HOSPITAL.—Miss Minnie Awcock has been appointed Nurse-Matron. She was trained at Brompton and Charing Cross Hospitals, and has since, for nine years, been matron at Newbury District Hospital.

### Minor Appointments.

THE INFIRMARY, EAST DULWICH GROVE.—Miss Maude Margaret Harwood has been appointed Night Superintendent. She was trained at the Hants County Hospital, Winchester, and was subsequently sister at the Eye and Ear Hospital, Southampton, and sister at the East Dulwich Infirmary.—Miss Ethel Gertrude Massey and Miss Agatha Leslie Laughlin have been appointed Sisters. Miss Massey was trained at the North Stafford Infirmary, Stoke, and has since been sister at the Royal Infirmary, Derby. Miss Laughlin was trained at the Royal Free Hospital, London, and has since been sister at the Royal Victoria Hospital, Bournemouth.

GLOUCESTER UNION HOSPITAL.—Miss Julia Lightfoot has been appointed Superintendent Nurse. She was trained at the Seamen's Hospital Society's Dreadnought Hospital, Greenwich. Subsequently she has held appointments at the Eastern Hospital, Homerton; the Seamen's Hospital Greenwich; and the Garrison Women's Hospital, Woolwich. She has also been district nurse at Hampton Wick, and Superintendent nurse at Cuckfield Union Infirmary.

MILITARY HOSPITAL, CORK.—Miss E. M. Woodman has been appointed Senior Sister. She was trained at the Royal Albert Edward Infirmary, Wigan, and subsequently served in the same institution for a year as sister. For three years and a half she has been head nurse at Professor Sinclair's Private Hospital, Manchester.

EAST POORHOUSE HOSPITAL, ABERDEEN.—Miss Bathia F. Davidson has been appointed Superintendent Nurse. She was trained for three years in the City Hospital, Aberdeen, after which she was in Smithstone Hospital, Greenock, as a Charge Nurse. Then for nine months she was a sister at the Cambridge Sanatorium, and since November last charge nurse at the East Poorhouse Hospital.

VICTORIA COTTAGE HOSPITAL, WOKING.—Miss A. B. Hall has been appointed Nurse She was trained for three years at the Royal Infirmary, Sheffield, and has since been engaged in private work at Cheltenham.

### Echoes from the Outside World.

### AN OPEN LETTER TO A HOSPITAL NURSE.

Our attention just now is more fully occupied with the official despatches published in the "Gazette" of Tuesday than with the advance of preparations for active measures at the front. Lord Roberts's kindness of heart is well known; how severely, and yet temperately, he can censure comes to most of us as a revelation. Even Sir Redvers Buller does not escape condemnation, because of his "disinclination to assert his authority, and see that what he thought best was done" in the matter of Spion Kop. The failure to hold this important post, according to the Field Marshal, was due as much to the former cause as to the "unwarrantable and needless assumption of responsibility" by Colonel Thorneycroft, who ordered the troops to retire. The last-named officer is, however, especially praised for his personal bravery. Sir Charles Warren and Sir Redvers Buller complain of each other, and Lord Roberts evidently considers that both of them in many ways occupy houses manufactured largely of glass, so that they are unable to blame each other. Several of the mistakes which are now recorded in black and white we had guessed at before, and I for one wish that despatches were not printed for the public. After all, Ladysmith has been relieved, and our dear "Bobs" is in command, two facts which go far to console us for any blunders committeed. This is far from being the first time that men of great capacity, with the best intentions, have erred, and assuredly it will not be the last.

As to the events of the week, the Queen contines to enjoy what she considers the rest and quiet of her Irish visit. For that reason she has declined to go to Belfast, very reluctantly, because she knows the disappointment it will give, but very wisely, because her health is so important to us all. Sir George White, strengthened and refreshed, has arrived in England, and his wife and daughter—who must often have wondered if they would ever see him again—met him at Southampton. Cronje with his wife and staff have arrived at St. Helena, from which fastness Colonel Schiel nearly succeeded in escaping, but the boatman inadvertently took his letter, making final arrangements, to H.M.S. "Niobe" instead of to the Dutch cruiser. At Ashanti the Governor and his wife and family are still kept prisoners by the rebels, and as yet there seems no sign of quashing the rebellion, though fresh troops have been dispatched to the Gold Coast, and the trouble is not looked upon as a serious one.

On Monday morning Mr. Chamberlain published an important communication from Sir Alfred Milner, which put into print and so made public a complaint which has been heard of for some time from private sources. I allude to the number of well-to-do folks who continue to flock to South Africa "for pleasure," a large proportion of whom are ladies. How a woman can find any "pleasure" in having the horrors of war brought more forcibly before her mind than they are by letters and the daily papers it is difficult to understand, and assuredly the plea that any part of Cape Colony or Natal, in its present overcrowded and necessarily insanitary condition, can be a "health resort," need not be discussed. Those on the spot assert, and I am afraid with a certain amount of truth, that many of the ladies are mothers with marriageable daughters, hoping for "better luck" where men are so plentiful than has attended their efforts in England. Others are women who live for excitement, and war is to them a new experience. Others, again, have relatives at the front and want to be near them. But perhaps the plain speaking of the High Commissioner will convince at least intending travellers of the widespread distress they are likely to cause if they persist in their plans. Prices in South Africa are always high; the more visitors who flock into the country the higher they will become, and the more suffering this will entail upon those whose business forces them, even with limited means, to remain abroad. When peace is once more restored, "personally conducted tours" round the battlefields for those who have a love of gruesome details is quite another matter. Meanwhile, the women of England can show their patriotism by remaining at home quite as strongly as the Boer women in the Transvaal are doing by being active. The late editor of a Pretoria journal says that he knows of as many as five hundred women in the artillery service of the forts around the capital, and that most females between the ages of eighteen and forty are armed. Fortunately, we do not need to fight, but we do need to render the conditions under which our men fight as good as they can be, and by wilfully shortening the supplies available at the base we are doing exactly the reverse.

A little time ago it was Mr. Winston Churchill who succeeded in being captured by the Boers and then escaped, thus providing himself with splendid "copy." Now it is Lord Rosslyn, whose enthusiasm for the drama has been succeeded by a desire to see the stirring scenes of real life at present being played upon the South African stage. He, too, having written in a very commonplace diary that he was starting on a long, tedious, uninteresting ride, with "just that element of risk of being 'nabbed' by the Boers which tempts me as an adventurous diary writer to go," departed with a kitbag, some luggage, and a servant, and promptly got himself taken by the enemy. He has been sent to Kroonstad, and will, I suppose, after a week or two succeed in making his escape and return to the English camp covered with pseudo-glory. Another scion of the nobility, Lord Brooke, the eldest son of the Earl of Warwick, is also to the fore. He seems a little young to have received a commission, and to have been allowed to join General French's staff, being only 17 years of age, whilst youths of the same age who wish to fight amongst the rank and file are considered too youthful to join, except as drummer or bugle boys. Therefore it is probable that, notwithstanding the "striking aptitude for a military career," which according to Sir Evelyn Wood he has shown, Lord Brooke will not as yet be given any very important duties which require training or experience.

The only novelty of note at the theatres this Easter has been the production at the Garrick of a version of a French play called "Zaza," which was written to enable Madame Réjane to show that she could invest a part of extreme vulgarity with a grace of her own. The American translation, which is represented by Mr. Charles Frohman's company, is understood to have been a great success in the United States, but the British public would not have been the losers if they had not been afforded the opportunity of recording their verdict. Nevertheless it would be rash to say that it will be unfavourable. The taste of a section of the community has become so vitiated that they will go to see and applaud anything that is sensational and deals with social problems which in more old-fashioned days were not discussed in the drawing-room. I am old-fashioned enough to take the earliest chance of indulging in a word of warning to those who may hear the moral of this meretricious piece and the acting lauded to the skies. It is quite true that vice is not in the end triumphant, and that Mrs. Leslie Carter impersonates the character of the flashy heroine with a certain measure of force. Her violence is certainly terrific, including china smashing, but of pathos she has none. And if she had, the most brilliant acting would not alter a story which deals from first to last with the so-called love of an opulent profligate for a semi-heathen music hall star. Of course, the former has a wife, for whom, equally of course, no sympathy seems to be necessary, and the clap-trap at the close does not invest the tawdry drama with any real charm. Nurses, I think, will not care to waste a portion of their little leisure in witnessing a performance which deals with disreputable Bohemianism in an unpleasant manner.

### Everybody's Opinion.

[Correspondence on all subjects is invited, but we cannot in any way be responsible for the opinions expressed by our correspondents. No communication can be entertained if the name and address of the correspondent is not given, as a guarantee of good faith but not necessarily for publication, or unless one side of the paper only is written on.]

### INEXPERIENCED MONTHLY NURSES.

"SISTER EVA" writes: If the blanket referred to by "A Sister" in your issue of April 7th could be depended upon for absolute cleanliness, the plan might be preferable. Experience, unfortunately, proves that this is seldom the case. There can be no surer way of transmitting puerperal septicæmia and other ills than by badly-washed blankets, and as these articles are usually sent to the general laundry, and are never boiled, I scarcely think they can be depended upon for the use to which Sister would put them. After a sanitary sheet has been cut into four pieces, and saturated with methylated spirit, it will burn easily enough in any fireplace.

### THE AGE LIMIT.

"PAVO" writes: As I have taken in your paper for years, and read everything, and take great interest in the discussions and letters, I most heartily thank Miss Gardner for her defence of nurses over thirty-five years, and if other ladies would help practically it would be a blessing to nurses; yet it seems a fashion to put, when advertising for a nurse, "Not over thirty-five years." Especially have I noticed this in regard to district nurses, who at this age should have their common-sense, self-reliance, and judgment matured. As very often a doctor cannot come in a minute, and a nurse has to act for the best alone, a nurse with a wider experience of life would be more helpful to a patient and friends than a younger one. Before Miss Mary Gardner's letter appeared I wondered what was to become of nurses and women generally over thirty-five years of age. If nurses will read and study and strive to find out new methods, I do not see why doctors should object to old nurses. I have gained a great deal of knowledge by reading the "Nursing Mirror" and THE HOSPITAL, and if a doctor is up-to-date I know how to go on, or to follow in his own way. I have managed to please doctors so far. I am surprised that more nurses have not thanked Miss Gardner, and I should have done so earlier, but thought that others more capable than I would do so.

### THE TITLE OF SISTER.

"ANOTHER NURSE" writes: I read with great interest your correspondent's ideas concerning the title of sister in a recent issue of the "Nursing Mirror." She writes, "There is great diversity in the training of nurses filling such offices." Here, I think, is the gist of the whole matter. Speaking generally, the title of sister is absolutely no indication of the stage of the nurse's career. Granted it should be; but of how many of our London hospitals can it truthfully be said the term "sister" is applied only to those nurses who have passed through the three or four years of training customary in their respective hospitals and obtained a certificate to that effect? In how many cases do we find the staff nurse working under the sister the more experienced woman of the two? How often does the conferring of the title "sister" depend mainly upon such external circumstances as a large income or influential connections? This, I know, is looking at the subject from rather a different point of view, one that your correspondent has not touched upon, but one which I venture to think is of far more importance than the somewhat vague possibility of the abolition of the mere title. Because the committee of a particular union refuse to confer the title of "sister" on their charge nurses, surely there can be no cause for fear that other, and more important institutions, will follow their example. I think I am correct in stating that there is not a single hospital in London without its sisters, nor any probability of their being abolished. It is most necessary that the woman at the head of the ward, who is responsible for the nurses working under her, and who has only the matron and doctors in authority over her, should have some distinctive title, just as she wears a uniform unlike the others. And "sister" being the most suitable in every way, will, in most quarters, no doubt continue to be, as it has always been, the title conferred on the nurse who is selected to occupy such a post.

### OUR WORKHOUSE INFIRMARIES—THE "ONE THING NEEDFUL."

"NURSE MARY" writes: Can anyone tell me if there is any justifiable reason for the following marked distinctions? In the greater number of our hospitals there is a permanent chaplain whose duty it is to care for the spiritual needs of the sick and dying; in addition to this, a short time is set apart morning and evening for a short, bright service. In our workhouse infirmaries this is not the case. Whilst much every way is being done to improve the system of workhouse nursing, and the bodily comfort of the patient is always the first consideration, yet no thought is given to the spiritual well-being of the patients beyond the visit of a clergyman sent for when a patient is said to be beyond recovery, and whom, on his arrival, is either too far gone to take part in any conversation or listen to any exhortation, if he has not already passed away. To my mind this is a question of vital importance. I was myself trained in a large workhouse infirmary of good standing and splendid organisation, and it has often been a source of great grief to me to see so many souls passing away into eternity without any concern being shown. Is it right that these people, for many of whom life has been one long struggle for existence, who have become hardened and embittered by circumstances, should be deprived of the opportunity of hearing the Gospel of our Lord Jesus Christ when they are most needing it, and when they would be more susceptible to its teaching? Are they not actually more in need of it than the more fortunate circumstanced ones in our big hospitals. Some of the objections I have heard are: "There is no time for it;" "A workhouse infirmary staff is limited, and the work heavy;" "There are so many Roman Catholics among the patients it would not be wise"—all good excuses in themselves, but do they justify this serious omission? To the first I would say, I have since been in hospitals where a quarter of an hour is devoted morning and evening to a short, bright service, and it has always been much appreciated. Although the patients have been a mixture of Church of England, Nonconformists, Jews, and Roman Catholics, it has in no way interfered with the usual routine, and I am convinced it has a good effect on the general behaviour of the patients. As to the other objection, why should the Roman Catholics be considered in this question? The priest is ever most assiduous in visits to his own flock, and no objection is raised. Would it be asking too much that they in return should maintain a respectful silence if not free to take part in a service contrary to their views? To my mind, no amount of bodily comfort can atone for the sad neglect of the souls of God's poor.

### To Nurses.

WE invite contributions from any of our readers, and shall be glad to pay for "Notes on News from the Nursing World," or for articles describing nursing experiences, or dealing with any nursing question from an original point of view. The minimum payment for contributions is 5s., but we welcome interesting contributions of a column, or a page, in length. It may be added that notices of entertainments, presentations, and deaths are not paid for, but, of course, we are always glad to receive them. All rejected manuscripts are returned in due course, and all payments for manuscripts used are made as early as possible at the beginning of each quarter.

### For Reading to the Sick.

GRANT Peace on earth,
And after we have striven,
Peace in Thy Heaven!
I do not ask, O Lord, that Thou should'st shed
Full radiance here;
Give but a ray of peace that I may tread
Without a fear.
Joy is like restless day; but Peace divine
Like quiet night;
Lead me, O Lord, till perfect day shall shine
Through Peace to light!

**Reading.**

My son, I have said, "Peace I leave with you, My Peace I give unto you: not as the world giveth, give I unto you."

All desire peace, but all do not attend to the things which bring true peace.

My peace is with the lowly and meek in heart; your peace shall be in much patience.

If you will hear Me and follow My voice, you shall be able to enjoy much peace.

What shall I do, Lord?

In everything give heed to yourself, both in what you do and in what you say, and always make it your aim to pleas Me only, and neither to desire nor to seek anything out of Me.—*Thos. A. Kempis.*

We have heard of the calm of an Indian summer. We have felt hours of stillness in the chequered, tossing light and shadow of the leaf-entangled forest in the quiet days of opening spring. We have listened to the drowsy hum of bees in the early summer in the fragrant sweetness of the swaying limes. We have known a sense of rest from weariness in the measured march of soothing music or the rise and fall of the heaving sea. Silent night has fallen upon us with unearthly calm after the restlessness of a weary day; the fret and trouble of working hours have sunk to stillness before the evening breezes and beneath the light of stars. Moments of quiet, whispers of rest! But these are external touches from nature or from man, sweet and consoling, comforting and tender. Not these, not these are the end of the soul's desire. Not even "the rest that remaineth," nor only the calmness of the calmness of the grave. No; the creature is yearning for the one blessedness, certainly in eternity; nay, even here.

The ground of the one real blessedness is a well-founded trust in Him to Whom he owes himself, his life, his all; the blessedness consists in an unbroken, a sustained communion with the Ever Blessed, with the living God.—*Canon Knox-Little.*

His will is our peace.—*Dante.*

Peace, perfect peace, with sorrows surging round?
On Jesus' bosom nought but calm is found.

Peace, perfect peace, with loved ones far away?
In Jesus' keeping we are safe and they.

Peace, perfect peace, our future all unknown?
Jesus we know, and He is on the Throne.

Peace, perfect peace, death shadowing us and ours?
Jesus has vanquished death and all its powers.

It is enough; earth's struggles soon shall cease,
And Jesus call us to Heaven's perfect peace.
—*Bickersteth.*

### Wants and Workers.

WILL some kind nurses send a little help to a poor invalid nurse, broken down in health from overwork, to assist her in furnishing a small bed-sitting-room? Her slender means are totally insufficient for this extra outlay. Contributions will be thankfully received for her by Miss M. E. Maingay, Havelet, Florence Road, Boscombe, Hants.

### Notes and Queries.

THE Editor is always willing to answer in this column, without any fee, all reasonable questions, as soon as possible.

But the following rules must be carefully observed:—

1. Every communication must be accompanied by the name and address of the writer.
2. The question must always bear upon nursing, directly or indirectly.

If an answer is required by letter a fee of half-a-crown must be enclosed with the note containing the inquiry.

*Maternity Training.*

(26) I should be glad to know of the best places to learn maternity nursing, and if I wished afterwards to try for the L.O.S. certificate, the easiest and most economical way? The York Road Hospital has not a vacancy so soon as I wished.—*C. A. G.*

The British Lying-In Hospital, Endell Street; the City Lying-in Hospital, City Road; and the East-End Mother's Home, Commercial Road, E., all offer good and cheap training in maternity nursing, and prepare pupils for the L.O.S. examination.

*Netley.*

(27) "E. R." would like to know how she could become a Netley nurse. She has had a year and seven months' training in a cottage hospital about twelve months ago, since then she has had private nursing.

"E. R." must first gain a three-year certificate from a general hospital before she is qualified for an army nursing sister. Apply then to the Under Secretary for State, War Office, Pall Mall, S.W.

*Training.*

(28) Will you kindly tell me if the training in midwifery is good at the Hospital for Women, Mill Lane, Woolwich, and how many beds there are? 2. I should also like to know if there is any reason why the same and the St. Mary's Nursing Home, Fulham, is not mentioned in the book, "How and Where to Train" (Burdett's)?—*Probationer.*

The Hospital for Women, Woolwich, is for soldiers' wives, and we do not know that it is a training school. 2. The St. Mary's Nursing Home is a private institution, and not managed by a committee. Therefore neither would be entered in "The Nursing Profession: How and Where to Train."

*Dispenser.*

(29) Will you kindly tell me what steps should be taken by a young lady wishing to become a dispenser? She has passed the first examination. Would it be advisable for her to be placed in a doctor's family where she could learn dispensing?—*A. M. B.*

Write to the Secretary, the Pharmaceutical Society of Great Britain, Bloomsbury Square, S.W., for information.

*Uniforms.*

(30) Could you kindly tell me what are the uniforms worn by the Sisters of the Army Nursing Reserve, Netley Sisters, and Indian Nursing Sisters, also Sisters and Probationers of Guy's, Bartholomew's, St. Thomas's, and the London? We are having a large bazaar in November in aid of funds for our new hospital, and it has been suggested that the stall-holders should wear uniforms of different hospitals.—*E.*

You will find illustrations of all the uniforms in "How to Become a Nurse," by Honnor Morten (Scientific Press). The dresses of the Army, Nursing Reserve are fully described in the "Mirror" of October 28th 1899, in "Notes on News," under the title "War Nurses."

*Fatherless Boy.*

(31) Will you kindly tell me if there are any homes for fatherless children where a boy 11 years of age would be finished in schooling and put to a trade or business. His mother is too poor to earn her living and bring up the child.—*S. E. S.*

There are a number such homes, and you would find the list in "Burdett's Hospitals and Charities" helpful (Scientific Press, price 5s.). The School of Handicrafts for Poor Boys (office, 32, Charing Cross) seems one of the most suitable for the case.

*Home for Incurable.*

(32) Can the Editor tell me of a home or institution where a lady of twenty-nine could be received free of any expense? She has been afflicted from her birth, and is perfectly helpless, but she is not imbecile. Her father, who has lately died, was a major in the army, and he has left no means for the support of his widow and family.—*Spes.*

The Royal Hospital for Incurables, West Hill, Putney Heath, S.W. (office, 106, Queen Victoria Street, E.C.), seems the charity most designed to help this kind of misfortune.

*Infirmary.*

(33) I should feel most thankful if you would kindly tell me whether it would be possible for me to get into a good general hospital after having been trained in a large infirmary (three years), or whether I shall have to go through probationership again for general work?—*E. R.*

If you have obtained your certificate you ought to have no difficulty in obtaining work. It is not every large hospital, however, which will accept infirmary-trained nurses.

*Standard Books of Reference.*

"The Nursing Profession: How and Where to Train." 2s. net.
"The Nurses' Dictionary of Medical Terms." 2s. 6d. net.
"Burdett's Series of Nursing Text-Books." 1s. each.
"A Handbook for Nurses." (Illustrated.) 5s.
"Nursing: Its Theory and Practice." New Edition. 3s. 6d.
"Helps in Sickness and to Health." Fifteenth Thousand. 5s.

All these are published by THE SCIENTIFIC PRESS, Ltd., and may be obtained through any bookseller or direct from the publishers, 28 & 29, Southampton Street, London, W.C.

### Travel Notes.

### XLVII.—FACILITIES FOR VISITING THE PARIS EXHIBITION.

The exertions made by tourist agents, hotel and pension keepers, and railway companies to meet the demands of the many thousands who will visit the Paris Exhibition of 1900 are so vast and comprehensive that they will be found to be suitable for the millionaire and for the poorest office clerk alike. It makes one's head swim to think of the labour and close calculation necessary to settle all these sliding scales of expense so accurately and yet leave a margin for profit to the originators.

### Independent Travel.

After close calculation I do not think anyone can go to Paris for a week and visit the Exhibition every day under £10, if they go on their own account. Not only will board and lodging be different to any other year, but cabs, guides, recognised tips, and the thousand and one other expenses that go to the making of a tour, will be largely increased in number and magnitude; therefore, my advice is, join either a personally-conducted tour set forth by one of the recognised agencies, or, at any rate, let them manage your business for you, and secure you accommodation.

### The Recognised Agencies.

The three agencies best known to the public are Messrs. Cook and Sons, Messrs. Henry Gaze and Sons, and Dr. Lunn's; all these three firms offer extraordinary facilities for seeing the Exhibition and Paris generally with the least possible outlay; they all also offer such tempting baits that it is extremely difficult to decide among their rival claims. I have had extensive dealings with all three firms, and have found them all equally courteous, liberal, and obliging. I think I shall help you best by telling you of some of the different conducted tours and tickets for independent travel offered by each.

### Messrs. Thomas Cook and Sons'

tours are numerous and tempting. First of all, they offer return tickets, third-class, from London Bridge to Paris from Saturday to Monday, with meat breakfast and dinner in Paris, and entrance to exhibition on Sunday, for 35s. This excursion is only fit for men; I consider it far too fatiguing for ladies, but it is excellent for those who can only reckon on the week-end. Next comes a list of conducted excursions which offer three days' third-class travel and three days' hotel accommodation for £2 11s. 9d.; four days for £3 5s.; and seven days for £4 11s. Second and first class are naturally more, but there is nothing against third-class travel on these excursions, because all classes travel by the same train. These tickets include (besides admission to the Exhibition) carriage drives round Paris for all but those who take third-class tickets for three days only. Of this class I should recommend that of third-class for seven days, £4 11s. A much more luxurious style of travel is offered in a tour lasting six days, everything first-class, and including three admissions to the Exhibition, rail and carriage excursion to Fontainebleau, and carriage drive to Versailles, all for £12. These are only a few of the offers made by Messrs. Cook, but my space being limited I must not mention more.

### Messrs. Gaze and Sons Offer boldly forty alternatives and ten prices. I will mention a few of them only. For one guinea you have a return ticket to Paris and one admission to the Exhibition, but naturally no hotel accommodation, so that you must go and return without sleeping, or pay separately. On the whole I do not think this so advantageous an arrangement as Messrs. Cook's Saturday to Monday offer for 35s., but on the other hand some of Messrs. Gaze's excursions are, if possible, still cheaper. For instance, No. 38 offers first-class return ticket, hotel accommodation for seven days, four admissions to the Exhibition, an excursion to the palace and forest of Fontainebleau, and drives to Versailles and round Paris, a five-franc seat at the Opera, and two tickets for special sights at the Exhibition, all for the sum of ten guineas. Among cheaper tours No. 5 for three guineas is very good. Return ticket third-class, hotel accommodation for three days, one admission to the Exhibition, and two drives, one of these being to Versailles. There is a great variety in these three-guinea tickets, no less than six, indeed. Those for four and five guineas differ in greater length of days and further drives. There are five different examples of tours for six guineas, No. 23 being a tempting one, and under the same heading is a cycling trip lasting nine days.

### Dr. Lunn's Arrangements.

Dr. Lunn makes some very tempting offers. His basis is that of a week's stay in all cases, except where travellers wish only a passing glimpse of the Exhibition on their way to Switzerland or elsewhere, when Dr. Lunn will meet their views, whatever they may be. The lowest priced excursion is five and a-half guineas. This includes second-class return ticket viâ Dover and Calais, and seven days' accommodation at a pension in the Champs Elysées, with six tickets of admission to the Exhibition. At first this appears much cheaper than other tours quoted above, but in reality it is about the same, because you will observe there are no drives or outside excursions. This would be an attraction to those who like to be as quiet as is consistent with a visit to such a place. Next comes the same length of visit, but costing seven guineas, the hotel accommodation being in the Hotel Castille, a good and well-established house, but to my mind too far from the Exhibition for those whose object in going to Paris is to visit the vast world's fair thoroughly. The most expensive tour costs ten guineas, and the tourists will stay at the Hotel d'Iena, close to the Trocadero and the Exhibition. The splendours of this palatial caravanserai will appeal to many who like to find themselves palatially lodged, and its position is splendid. For myself I prefer the modest Pension Robin.

### Impedimenta for the Trip.

Let me entreat you—as one who knows and has suffered—not to burden yourself with unnecessary garments. Believe me, nobody in that vast throng will ever look at your clothes provided they are neat and suitable. Take nothing that is crushable and no washing dresses; they become all crumpled and dowdy in a crowd.

### TRAVEL NOTES AND QUERIES.

Rules in Regard to Correspondence for this Section.—All questioners must use a pseudonym for publication, but the communication must also bear the writer's own name and address as well, which will be regarded as confidential. All such communications to be addressed "Travel Editor, 'Nursing Mirror,' 28, Southampton Street, Strand." No charge will be made for inserting and answering questions in the inquiry column, and all will be answered in rotation as space permits. If an answer by letter is required, a stamped and addressed envelope must be enclosed, together with 2s. 6d., which fee will be devoted to the objects of the "Hospital Convalescent Fund." Any inquiries reaching the office after Monday cannot be answered in "The Mirror" of the current week.

The Seine (Cyclist).—Yes, it is a delightful cycling ground, not very hilly, and with plenty of variety. You might go viâ Dieppe, then take the train to Rouen or cycle if you prefer it. The distance is about forty miles, or rather less; having explored the neighbourhood of Rouen and visited Evreux, and perhaps the two Andelys, you can start down the Seine. I should advise Caudebec as your head-quarters.

Belgium (Inquirer).—You can have a fair idea of the more important parts of the country in a month, but it will be hard work and you will miss much of interest. Here is a skeleton tour with that object in view. Begin at Antwerp, Mechlin, Brussels (from this last you can go to Louvain for the day), Alost, Ghent, Ypres (from which visit Courtrain and Audenard), finishing with Bruges. From there you will return to England viâ Ostend. You can do this for £17.

Bregenz—Is it cheap? (Erl King).—I think for an artist of the stronger sex, living may be very reasonable, four and sixpence or five shillings a day. It is full of artistic merit, and excursions all round are plentiful.

